# The string of pearls pattern in dermoscopy of a chest papule^☆^^☆☆^

**DOI:** 10.1016/j.abd.2019.06.015

**Published:** 2020-03-20

**Authors:** Li-Wen Zhang, Wen-Ju Wang, Cong-Hui Li, Tao Chen

**Affiliations:** Department of Dermatovenereology, Chengdu Second People's Hospital, Chengdu, Sichuan, China

Dear Editor,

A 49-year-old woman presented with a 3-year history of an asymptomatic red dome-shaped papule on her left chest. The lesion slowly enlarged (Fig. 1). There was no relevant medical history and family history. Dermoscopy showed glomerular and dotted vessels with “string of pearls” pattern distribution, and a collarette of scale surrounding (Fig. 2). Excisional biopsy of the lesion with histopathological examination showed a sharply demarcated psoriasiform epidermal hyperplasia composed of slightly enlarged clear keratinocytes and basal cells with pale-staining cytoplasm, dilated blood vessels in the dermal papillae as well as perivascular mixed inflammatory infiltration (Fig. 3). We diagnosed this patient as having clear cell acanthoma (CCA), without recurrence for 4 months after excision.

**Figure 1:**
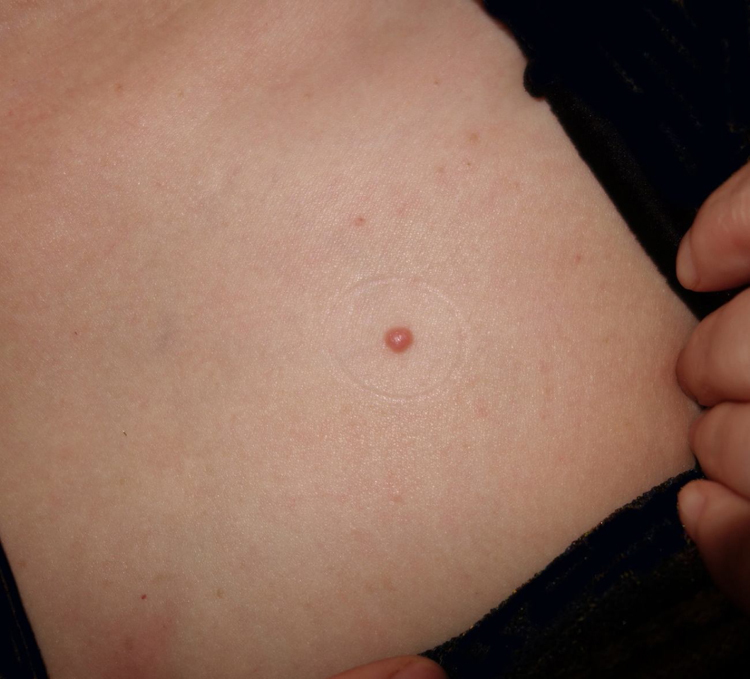
Solitary papule on her chest.

**Figure 2:**
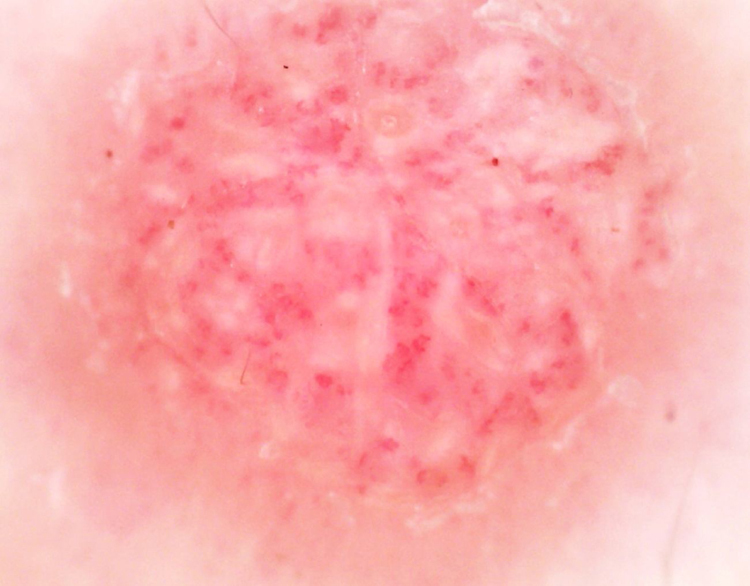
Dermoscopy showing glomerular and dotted vessels with “string of pearls” pattern distribution and a collarette of scale surrounding.

**Figure 3:**
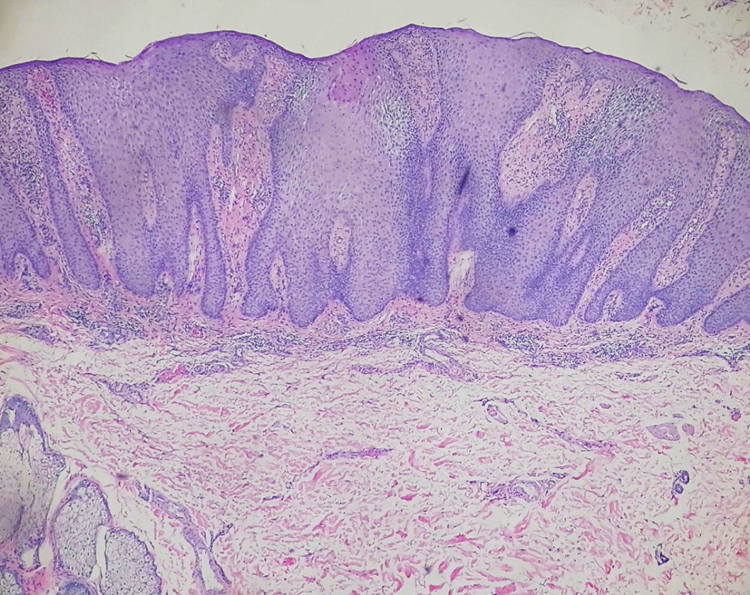
Histopathology showing a sharply demarcated psoriasiform epidermal hyperplasia composed of clear keratinocytes and basal cells with pale-staining cytoplasm, dilated blood vessels in the dermal papillae as well as perivascular mixed inflammatory infiltration (Hematoxylin & eosin, ×40).

CCA, or Degos acanthoma, is a rare clinical entity first described by Degos in 1962.^1^ The etiology of CCA is still not clearly known. It is traditionally considered to be a form of benign epidermal neoplasia. Meanwhile, there are speculations that CCA may be an inflammatory reactive dermatosis or localized psoriasis.^2^ CCA generally arises on the lower extremities and is characterized by an asymptomatic, red-brown, dome-shaped, solitary papule or nodule often covered by a thin collarette of scale. Additionally, giant, pedunculated, multiple, cystic and pigmented patterns may be seen in CCA. The histopathological features include a well-demarcated area of psoriasiform acanthosis with keratinocytes with pale staining cytoplasm, intercellular edema, parakeratotic microabscesses and edematous papillary dermis as well as the superficial capillaries and veins increased. CCA must be differentiated from actinic or seborrhoeic keratoses, basal cell carcinoma, squamous cell carcinoma, pyogenic granulomas, clear cell hidradenoma and amelanotic melanoma.

Under dermoscopy, CCA has a unique appearance that is helpful to improve diagnostic accuracy, characterized by red dots, globules, and glomerular vessels arranged in linear or serpiginous patterns as well as a collarette of scale surrounding.^3^ The dermoscopic feature of “string of pearls” corresponds to the histopathology of CCA on transverse sections that indicates uniformly sized dermal islands with a string-like distribution and abundant dilated capillaries.^4^ However, Espinosa recently reported that the dermoscopic feature of “string of pearls” pattern was also found in seborrheic keratosis and lichen-planus-like keratosis and suggested that other epidermal tumors, especially having the similar pathological pattern, could also show this dermoscopic pattern.^5^ Hence, Biopsy is still not avoided in the diagnosis of CCA.

## Financial support

None declared.

## Authors’ contributions

Li-wen Zhang: Conception and planning of the study; obtaining, analysis, and interpretation of the data; critical review of the literature.

Wen-Ju Wang: Concepção e planejamento do estudo.

Cong-Hui Li: Critical review of the literature.

Tao Chen: Critical review of the manuscript.

## Conflicts of interest

None declared.
